# Quantitative Analysis of Cell Migration Using Optical Flow

**DOI:** 10.1371/journal.pone.0069574

**Published:** 2013-07-31

**Authors:** Katica Boric, Patricio Orio, Thierry Viéville, Kathleen Whitlock

**Affiliations:** 1 Centro Interdisciplinario de Neurociencia, Facultad de Ciencias, Universidad de Valparaíso, Valparaíso, Chile; 2 Inria, Mnemosyne, Sophia Antipolis, France; School of Biomedical Sciences, The University of Queensland, Australia

## Abstract

Neural crest cells exhibit dramatic migration behaviors as they populate their distant targets. Using a line of zebrafish expressing green fluorescent protein (*sox10:EGFP*) in neural crest cells we developed an assay to analyze and quantify cell migration as a *population*, and use it here to characterize in detail the subtle defects in cell migration caused by ethanol exposure during early development. The challenge was to quantify changes in the *in vivo* migration of all Sox10:EGFP expressing cells in the visual field of time-lapse movies. To perform this analysis we used an Optical Flow algorithm for motion detection and combined the analysis with a fit to an affine transformation. Through this analysis we detected and quantified significant differences in the cell migrations of Sox10:EGFP positive cranial neural crest populations in ethanol treated versus untreated embryos. Specifically, treatment affected migration by increasing the left-right asymmetry of the migrating cells and by altering the direction of cell movements. Thus, by applying this novel computational analysis, we were able to quantify the movements of populations of cells, allowing us to detect subtle changes in cell behaviors. Because cranial neural crest cells contribute to the formation of the frontal mass these subtle differences may underlie commonly observed facial asymmetries in normal human populations.

## Introduction

The cranial neural crest cells (CNCC) are a population of progenitors that give rise to both craniofacial structures and components of the peripheral nervous system (PNS). These cells migrate from the dorsal neural tube to populate the face. After leaving the neuroepithelium, CNCC split into discrete streams separated by CNCC-free regions [1]. Three main streams are formed according to their relative pre-migratory position along the cranial neural tube. The most anterior stream of CNCC migrates dorsal to the eye and populates the frontal mass (or anterior neurocranium) [2], ganglia of cranial nerves and melanocytes among others. More posterior streams of CNCC populate the branchial arches, where they form the cartilage and bones of the jaw [3]. Much is known about the CNCC migration and differentiation as it relates to the branchial arch derivatives including the jaw elements [4], [5]. By contrast, the migration of CNCC that pass dorsal to the eye and contribute to the facial structures has not been extensively described with the exception of the migration of neural crest cells that contribute to specific parts of the neurocranium [2], [6], [7]. We (this study) and others [8] have found that the migration of the dorsal anterior CNCC appears to be sensitive to environmental factors such as alcohol, smoking, and herbicides. Yet, the cell movements that occur during the migration of this population of CNCC are complex and difficult to describe and characterize quantitatively. For this reason we developed an analytical method to characterize cell migration, and use it here to describe the defects of dorsal anterior CNCC migration caused by alcohol exposure.

There is a large body of evidence demonstrating that ethanol (EtOH) affects CNCC development. The primary defects observed are cell death in both *in vitro* and *in vivo* systems; some studies also report defects in cell migration. *In vivo* EtOH-induced cell death has been reported in regions populated by CNCC in the developing chick embryo [9] and while the number of CNCC is reduced, the migration patterns were unaffected [10]. Additional studies in chick have reported cell death in neural crest cells [11]. *In vitro* analysis of CNCC has shown that EtOH exposure produces permanent changes in cell shape, surface morphology, migration, and cell death [12], [13]. Interestingly no analysis of migration defects have been reported *in vivo*, although histological studies have suggested the occurrence of EtOH-induced migration defects, based on the pattern of immunostained neural crest in treated chick embryos [14]. Thus, whereas EtOH induced cell death has consistently been reported in a variety of model systems, the effects on CNCC migration have not been well documented. An *in vivo* real time analysis of the CNCC population contributing to the frontal mass would improve our understanding of CNCC migration in normal and EtOH exposed animals.

Many studies of cell migration use techniques that track single cells and base their analysis on the average migration behavior of single cells. In zebrafish and *Xenopus* embryos the cell-cell interactions of individual CNCC have been analyzed to demonstrate contact inhibition of locomotion in which cells cease migrating after contact with another cell [15]. Because the CNCC undergo “collective cell migration” where the cooperation between cells contributes to the overall directionality of the group [1], it is important to analyze the migration of the CNCC as a population. However, programs for this application have not been developed. Optical Flow (OF) is an image processing method that can track motion in image sequences, and is thus potentially useful for tracking populations of cells. OF computation methods have been used in computer and vision sciences (reviewed in [16]) but have also been used in the life sciences to quantify cell and tissue movements of *Dictyostelium* [17], [18], to track cell behaviors during *in vitro* wound healing [19], and most recently to study trafficking of GABA receptors into dendrites of hippocampus neurons [20]. An advantage of zebrafish as a model system is that the migration of cells can be studied in wholemount *in vivo* systems but, to date, OF analysis has not been used to study cell migration in zebrafish embryos. Our *in vivo* analysis of CNCC using the OF methodology allows for a population-wide analysis of cell migration in the early embryo. This type of analysis can detect the subtle changes induced by EtOH, which are difficult to quantify, and are potentially relevant for understanding EtOH induced phenotypes [8].

## Materials and Methods

### Fish Maintenance

Zebrafish (*Danio rerio*) were maintained on a 14/10 hours light/dark cycle and raised under standard conditions [21]. Experiments using wild-type embryos were done using the “Cornell” strain, derived from the AB strain (University of Oregon) and pet store fish in the Whitlock lab. The line *sox10:EGFP* [2] was provided by the Kelsh Lab. This study was carried out in strict accordance with the recommendations in the Guide for the Care and Use of Laboratory Animals of the National Institutes of Health. The protocol was approved by the Committee for Ethical Use of Experimental Animals, Universidad de Valparaíso (Project Approval # 111046). No invasive experiments were performed and all efforts were made to minimize suffering.

### Ethanol Exposure

The ethanol exposure protocol was carried out according to a previously published study in zebrafish [22], where exposure started at 4 hours post-fertilization (hpf) and embryos were grown to specific developmental stages dependent upon the analysis. Embryos (n = 80/experiment) were maintained in glass Petri dishes (100×15 mm) containing 30 ml of embryo medium [21] alone (control) or with 100 or 200 mM EtOH concentrations (C_2_H_5_O, 99.9% Absolute ethanol for analysis, stock # K41470083 040, Merck, Germany). Dishes were sealed with parafilm to prevent EtOH evaporation. The concentrations were chosen because they are sublethal, with the majority of the exposed embryos growing to adulthood.

### Cell Death Detection

Cell death was assessed using the TUNEL based *In Situ* Cell Death Detection Kit, (TMR red, Roche, Switzerland) in control and treated 24 hpf *sox10*:*EGFP* embryos and processed according manufacturer’s protocol. An observer blind to the experiment counted TUNEL positive cells in the craniofacial area and determined whether they also expressed Sox10:EGFP.

### Time-lapse Imaging

*In vivo* analysis of the anterior CNCC migration was performed in *sox10:EGFP* embryos. The embryos were mounted using 1.5% low-melting agarose (Sigma-Aldrich) in an Attofluor chamber (A-7816, Invitrogen), containing either embryo medium alone (control) or embryo medium with 100 or 200 mM EtOH (0.6–1.2% v/v). Embryos were imaged using a Spinning Disc confocal microscope Olympus BX-DSU (Olympus, Japan). Movies captured 6 hours of development and were initiated at 6–8 somites stage and terminated at 20–22 somites stage. Time-lapse movies for Optical Flow analysis were generated by collecting *Z*-Stacks (30 images every 1 *µ*m) every 7.5 minutes using an ORCA IR2 Hamamatsu camera (Hamamatsu Photonics, Japan). Images were acquired and processed using the Olympus Cell-R software (Olympus Soft Imaging Solutions, Germany) and processed using AutoQuantX 2.2.2 (Media Cybernetics, USA) deconvolution software. For each treatment (control, 100 mM, 200 mM) five samples were analyzed using OF.

### Migration Analysis: Optical Flow and Affine Flow

**Figure 1:**
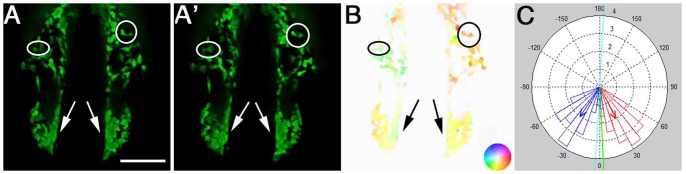
Optical Flow analyses of net cell movement. A, A’) Two consecutive frames of a time-lapse movie. B) Optical Flow analysis yields a vector field that is represented in a color-coded manner. As shown in the inset, color hue represents direction of movement and color intensity represents magnitude of movement. C) Polar histogram showing the distribution of the summed angles of motion vectors detected by OF. Blue and red bars depict binned movement in the left and right side of the field, respectively. Red and blue arrows represent the average vector of movement, and the green arrow is the average vector over the whole field. Scale Bar = 50 µm. Anterior movements are toward the bottom of the page.

The migratory behaviors of CNCC populations captured in time-lapse movies (Figure 1A A’ for example) were quantified using the Optical Flow (OF) image processing method. OF analysis was performed using the algorithm described in [16] that combines global and local fitting approaches. OF procedures were executed in Matlab R2011b (MathWorks, Natick, MA, USA) using a freely available code (http://people.csail.mit.edu/celiu/OpticalFlow/) that implements the Bruhn/Weickert/Schnörr equations [23]. An additional Matlab code was written for further analysis and plotting of data.

OF finds the motion vectors 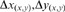 that best describe the differences in intensity between two consecutive images; such that:
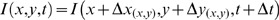
where 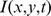
is the intensity of the pixel located at 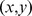
in the frame corresponding to time 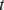
_,_ and 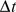 is the time interval between two frames. Thus, the OF analysis calculates how much each position or pixel 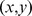 in the image has moved from one image to the next. The result is a *vector field*, where for each pixel in the image there is a vector (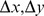) that represents the displacement of that given pixel. With this processing, the CNCC migration can be described as lateral 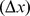
and vertical (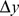) displacements, or as angle (θ) and magnitude (*r*) of movements. The resulting vector field is represented as a color coded image in which color intensity represents movement magnitude and a given color hue represents the movement’s direction (see Fig. 1B for an example). For all analyses described in the Results section, vectors in the OF frames were considered only for pixels whose intensity in the first image was equal or higher than 2.5 of the mean image intensity. This eliminated movement vectors assigned to dark areas of the image.

In order to compare the horizontal 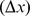 movements, we divided the vector field into left and right fields by the vertical center of the image (see polar graph in Fig. 1C). Then, the 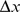
components of vectors in the *left* field were inverted in sign, thus a positive value in either the left or right field means an expansive movement (towards the lateral edges of the field) whereas a negative value means a contractive movement (towards the center).

#### Angle statistics

We calculated the angle (θ) and magnitude (*r*) of cell movements and averaged them in a per-frame basis. Also, vector fields were divided in left and right from the center of the image and the average vector was calculated for each half of the field, for each OF frame. In each case the mean vector of each side was calculated as:
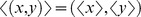
where the angled straight brackets denote the mean.

#### Affine transformation fit

The 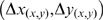 vector fields were fit to the linear functions:
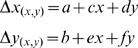



These allow the characterization of the cell movements in terms of position-independent components (*a*, horizontal; *b*, vertical) plus the position-dependent coefficients that quantify expansion-contraction (*c*+*f*). As the Affine transformation tries to capture the mean behavior of the OF field, outlier vectors have to be disregarded as much as possible. To accomplish this, Affine fitting was performed to a moving average of 5 OF frames, thus a sequence of *n* OF frames yields *n*-4 Affine frames. The fit was performed using a RANSAC (Random Sample Consensus) method where the fit is performed to a random sub-sample of the data and the number of inliers to that fit is determined using some distance criterion. The fit is repeated over many random sub-samples and the largest set of inliers is always kept, iterating until the probability of finding a larger inliers set is very low. The final fit is then performed to the largest set of inliers found.

### Statistical Analysis

All statistical analyses were performed using GraphPad Prism 4.0 (GraphPad, USA).

## Results

### Quantifying Population Migration of CNCC in Control and EtOH Treated Embryos

**Figure 2:**
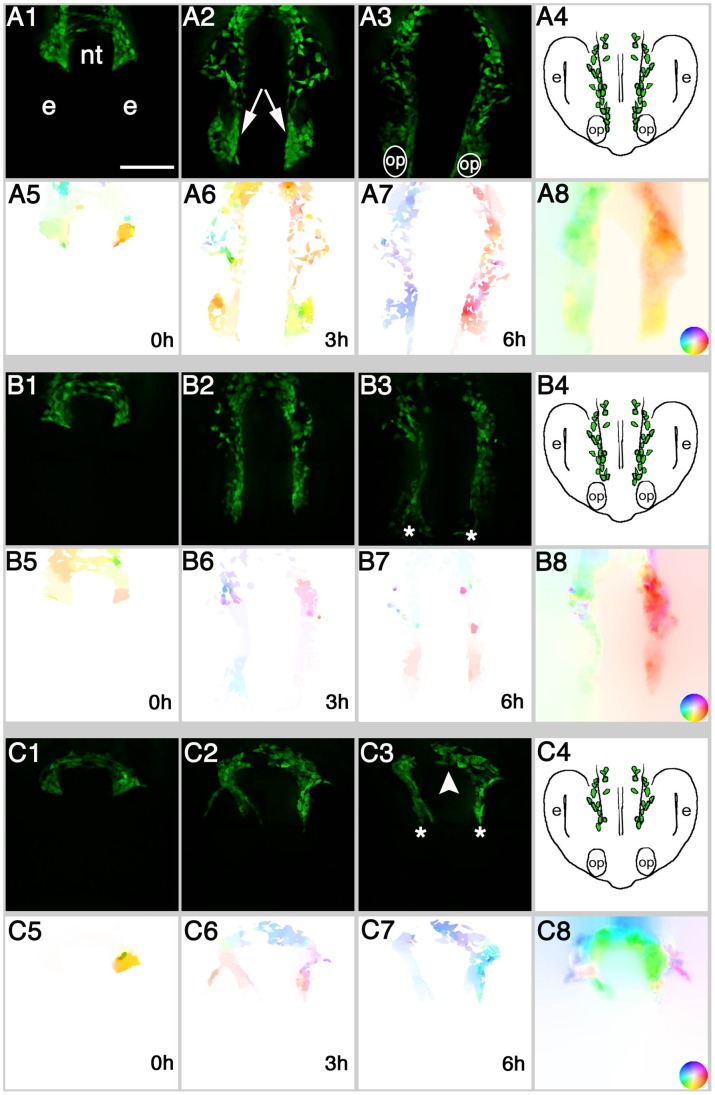
Analysis of cell migration at the population level reveals EtOH induced differences in CNCC migration patterns. Representative examples of A) Control, embryos treated in B) 100 mM and C) 200 mM EtOH. 1–3) Video frames captured from time-lapse movies at beginning (0 h) middle (3 h) and end (6 h) of the recording period. 4) Cartoon of a 20–22 somite embryo, showing location of CNCC (green) at the end of migration. 5–7) Color-coded image showing the movement vectors detected by OF analysis. 8) Movement vectors summed for the complete six-hour sequence. The inset is the color scale representing direction and magnitude of movement. Color intensity represents movement magnitude and a given color hue represents the movement’s direction. Dorso-Frontal views: posterior to the top. Scale Bar = 50 µm. nt = neural tube, e = developing eye, op = olfactory placode. Anterior movements are toward the bottom of the page.

In order to analyze CNCC migration on a population-wide basis we performed *in vivo* time-lapse movies, using the transgenic line *sox10:EGFP*. The movies were started at the 6–8 somite stage and continued until the 20–22 somite stage. During this six-hour window CNCC moved anteriorly to populate the frontal mass. In control embryos, at time 0 (6–8 somites), the CNCC were located dorsal-posterior to the eye (Fig. 2A1, see Video S1) and moved anteriorly as two groups of cells lying between the neural tube and the inner edge of the eye. Three hours after initiation of the time-lapse the cells were adjacent to the medial edges of the eyes (Fig. 2A2, arrows) and at the end of the movie (20–22 somite stage) the cells reached and surrounded the olfactory placodes (OPs) (Fig. 2A3, circles), as reported previously [24]. In contrast, CNCC in embryos exposed to 100 mM EtOH (Fig. 2,B1,2,3; see Video S2) started migrating anteriorly in a similar manner but appeared to advance less (Fig. 2B2) than in the control embryo (Fig. 2,A2, arrows). By the 20–22 somite stage (end of time-lapse), the cells had reached the posterior limit of the OP (Fig. 2,B3, asterisks) and appeared less organized compared to the controls (Fig. 2A3). In embryos exposed to 200 mM (Fig. 2C1,2,3, see Video S3), the effects of EtOH on CNCC migration were more severe: the CNCC start migrating anteriorly (Fig. 2 C1,2), though some cells failed to move (Fig. 2C3, arrowhead) and by 20–22 somites the cells had arrested, never reaching the limits of the OPs (Fig. 2C, asterisks). Thus, exposure to sublethal concentrations of EtOH resulted in a qualitative effect on the migration of CNCC; the challenge was to then quantify these effects.

#### Global analysis of cell movements

Using OF, we analyzed movies of CNCC migration patterns over time in control and EtOH treated embryos. The CNCC migration can be described by lateral and vertical pixel displacements, with observed angle and magnitude of movements resulting in a vector field in which color intensity represents movement magnitude and a color hue represents the movement’s direction (Fig. 2). The movement analysis (performed on the whole sequence) is shown for three time points: 0, 3 and 6 hours, as well as a sum of the whole sequence data (Fig. 2A8, 2B8, 2C8). In the control embryos (Fig. 2A5–7), horizontal cell movements were observed in the two bilateral groups of cells moving laterally and in opposite directions, one side moving left (Fig. 2A8, green-blue colors) and the other moving right (Fig. 2A8, orange-red color). The posterior-anterior CNCC movements showed a net anterior movement over time (Fig. 2A8, yellow).

Analyses of 100 mM EtOH treated embryos (Fig. 2B5–7) revealed that the horizontal cell movements also followed a left and right pattern over time (Fig. 2B8, green, red) similar to that observed in control embryos (Fig. 2A8). The posterior-anterior cell movements in these embryos also showed a net anterior movement over time (Fig. 2B8, yellow). In contrast, the embryos treated with 200 mM EtOH showed a greater range of horizontal cell movements, as evidenced by the increased distance between the two bilateral groups of cells (Fig. 2C8) compared to that in control embryos (Fig. 2A8). Horizontal cell movements were more scattered as shown by the increase in color hues within the groups of cells (Fig. 2C8). Moreover, the cells from the right side were moving left, as shown by the green colored cells (Fig. 2C8). Interestingly, the CNCC in these embryos showed movements toward the posterior region (Fig. 2C8, blue-magenta colors), which is one of the more extreme phenotypes we observed in EtOH treated embryos.

#### Analysis of averaged cell movements

**Figure 3:**
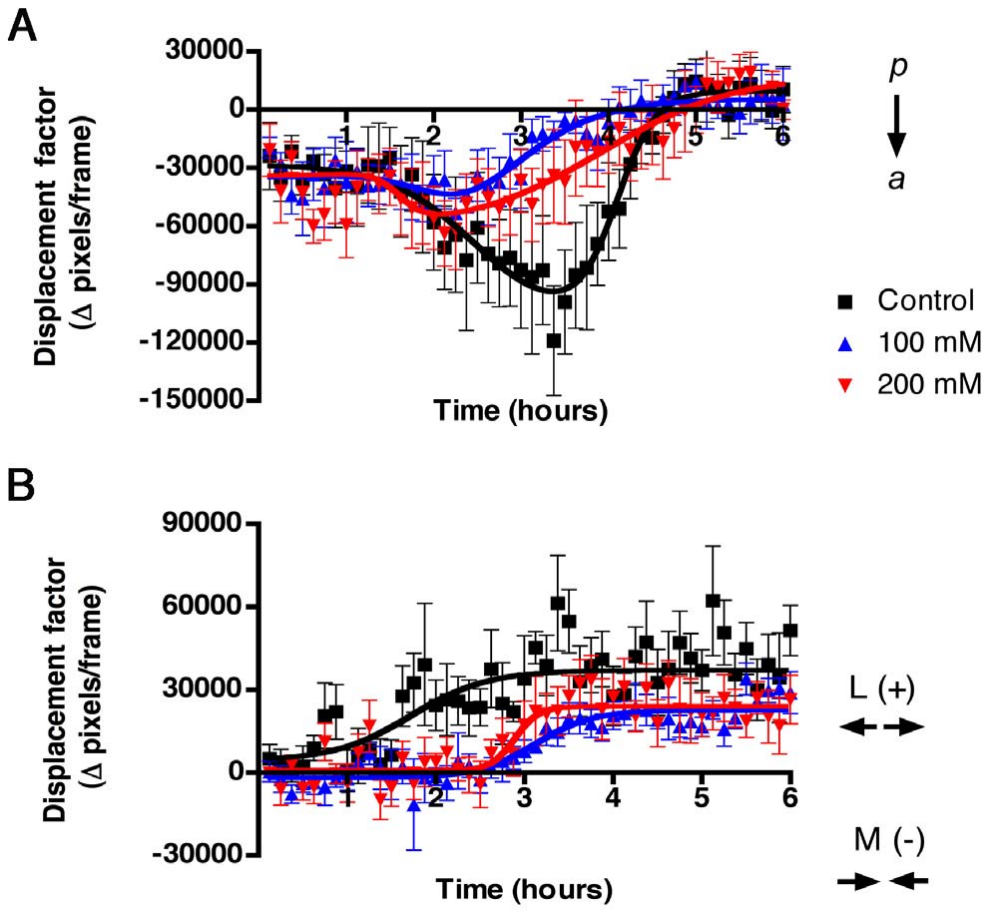
Optical Flow averaged cell movements are different in EtOH treated vs. control groups. OF analysis were averaged and represented over time as net displacement reflecting: A) Posterior-Anterior cell movements over time (negative numbers in the Y-axis represent anterior cell movements) and B) Horizontal cell movements (medial and lateral) over time (negative numbers in the Y-axis represent movements towards the middle of the field). Lines show the sum of two sigmoid functions. n = 5 per group. a = anterior, p = posterior, L = lateral movements M = medial movements. Error bars = SEM.

In order to quantify the CNCC movements through time in the control vs. the EtOH treatment groups (n = 5 each); posterior-anterior (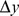) and horizontal (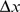) movements were summed for each frame of the OF sequences and plotted against time. The average posterior-anterior migration behavior of CNCC per frame was plotted as a displacement factor over time (Fig. 3A). At the beginning of migration (from 0 to the first hour), control (Fig. 3A, black), 100 mM (Fig. 3A, blue) and 200 mM EtOH (Fig. 3A, red) showed a similar anterior displacement. In contrast, during the second hour the control group displayed larger anterior displacement than the treated groups, reaching a maximum at 3–4 hours. Both 100 and 200 mM treated groups reached their maximum anterior displacement before the control group, between the second and third hour (Fig. 3A). At 4.5 hours the CNCC in controls no longer showed a net forward displacement, which is reflected in the graph by the curve returning to values near to zero. The lines in the graph depict a fit to the sum of two sigmoid functions that allows the time point in which the cells cease to move to be estimated. The control group shifted its direction of cell movements at 4.3 hours, the 100 mM group did so at 3.2 hours and the 200 mM at 3.9 hours. Thus, CNCC in both treated groups decreased their net displacement before those in the control group. Interestingly, the shallower slope of the fits shown in both treated groups (Fig. 3A, blue, red) indicates that the decrease of posterior-anterior movement occurred in a less concerted fashion than in the control group (Fig. 3A, black). In order to perform statistical analyses on these results (Fig. 3A), we binned the data by time periods and compared the group results every hour with a variance analysis (ANOVA). No significant differences between the groups were found at the first and fifth hours, whereas significant differences among groups were found during the second, third, fourth and sixth hours (p<0.0001 for all cases, Table 1).

**Table 1:** Summary of p values obtained in the ANOVA of the different OF and affine parameters.

Hours	OF PA^1^	OF HOR^2^	affine E-C^3^	affine PA^4^
1st	0.0948	0.0016	0.7842	0.0987
2nd	0.0001	0.0012	0.0001	0.0001
3rd	0.0001	0.0001	0.0001	0.0001
4th	0.0001	0.0001	0.2005	0.6265
5th	0.1204	0.0001	0.0001	0.0068
6th	0.0001	0.0001	0.0001	0.2292

1 from Fig.3A.

2 from Fig.3B.

3 from Fig.5A.

4 from Fig.5B.

In order to compare the horizontal movements, we divided the vector field into left and right fields by the vertical center of the image. Then, the 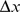 components of vectors in the *left* field were inverted in sign, thus a positive value in either the left or right field means the cells moved laterally relative to the midline of the field whereas a negative value means the cells moved medially (towards the center of the field). The values were summed for each frame and the averages for the experimental conditions were plotted as displacement over time (Fig. 3B). In all groups, the cells started moving horizontally, but, as they migrated, the controls (Fig. 3B, black) showed an increase in lateral cell movements in comparison to both EtOH treated groups (Fig. 3B,blue, red). Again, data were binned by time periods and ANOVA yielded significant differences among groups through all time periods (Table 1). Thus, EtOH alters the pattern of CNCC migration by significantly reducing cell movements over time. The cells of EtOH treated embryos migrate less in both anterior and lateral directions in comparison to control embryos and it takes them longer to transition between types of movements (transition from posterior/anterior to outward movement).

#### Analysis of net movement and directionality

**Figure 4:**
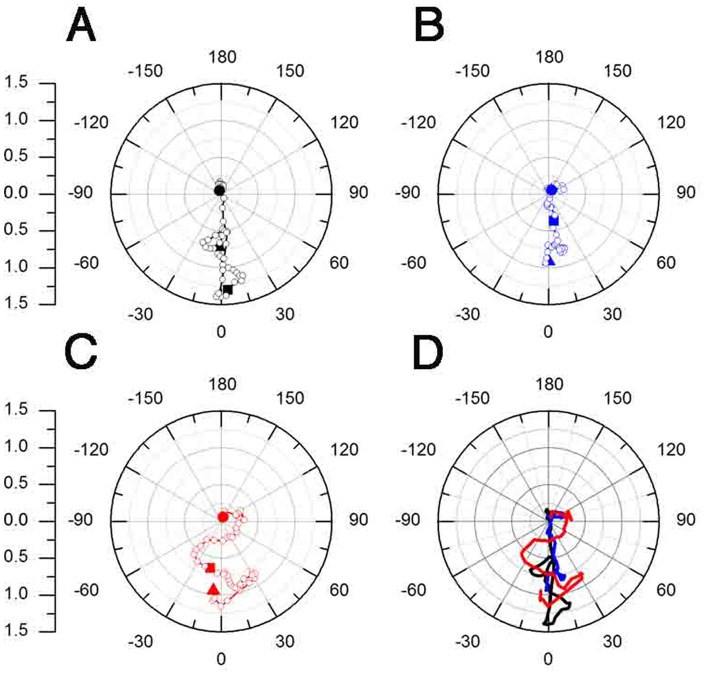
Ethanol alters CNCC net movement and directionality. A–D) Polar graphs of the angle (q) and magnitude (*r*) of the mean motion vector over time. A) Control showing net anterior movement of CNCC. B) 100 mM EtOH showing reduced magnitude but net anterior movement. C) 200 mM EtOH showing both reduced magnitude and loss of directionality. D) Summary of A–C. The triangles indicate migration at t = 0, squares t = 3 h, and circles at t = 6 h. Open circles represent individual data points during the sequence. The scale on left shows the magnitude (*r*) values.

**Figure 5:**
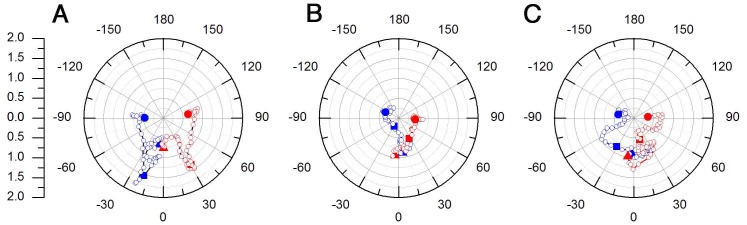
Ethanol alters CNCC lateral movement directionality. A–C) Polar graphs of the lateral angle and magnitude (*r*) of the mean motion left vector (blue) and right vector (red) over time. A) Control showing symmetrical left-right movements. B) 100 mM EtOH treatment has reduced magnitude and less symmetrical left-right movements. C) 200 mM treatment has both reduced magnitude and asymmetrical left-right movements. The triangles indicate the results for the first sample, the squares those after three hours and the circles those after six hours. The open circles are each time point during the sequence. The scale bar in the left shows the magnitude (*r*) values.

To obtain a more complete picture of the differences between control and treated embryos we characterized the pattern of CNCC migration by calculating the mean motion vector of each frame and plotting its angle (θ) and magnitude (*r*). The motion vectors were averaged for each frame of the OF sequences and plotted as polar graphs (Fig. 4 and 5). In these plots, each symbol represents the tip of a vector that originates at the center. The first mean motion vector (t = 0 h) is shown as a triangle, the mean motion vector of the middle of the OF sequences (t = 3 h) is represented by a square and the last motion vector is depicted by a circle (t = 6 h). The control group (Fig. 4A) showed a very directed anterior migration of cells over time, given by the position of the majority of the vectors around the 0° axis. The magnitude of the vectors (distance from the center of the graph) increased during the first three hours of migration as evidenced by the fact that the first sample vector (Fig. 4A, t = 0 h, triangle) had a magnitude of approximately 0.7 (Fig. 4A, triangle), which increased to 1.3 at three hours (Fig. 4A, t = 3 h, square). Overall, cells increased their movements in the anterior direction during the first 3 hours of migration. In the last hours of recording, the vectors, including the last sample vector (Fig. 4A, t = 6, circle), showed a reduction in magnitude. This apparent reduction in magnitude at the end of the migration occurred because the cells move laterally, thereby cancelling the net movement when averaging over the whole frame (see below, Fig. 5).

The 100 mM EtOH (Fig. 4B) treated group also showed an overall anterior directed migration, as evidenced by the grouping of the vectors around the 0° axis. However, the first sample vector had an approximate magnitude of 1 (Fig. 4B, t = 0 h, triangle) whereas the vector sample at three hours (Fig. 4B, t = 3 h, square) had a 0.5 magnitude. Thus the magnitude of the vectors decreased during the first three hours of migration.

In the 200 mM EtOH treated group (Fig. 4C) the anterior cell movement appeared less coordinated, as evidenced by the vectors crossing the 0° axis several times (Fig. 4C) towards −30° and 30°. As in the 100 mM group, the magnitude of cell movements was decreased when compared to that of the control group, as shown by the decreased magnitude of the vectors during the first three hours of migration. The first vector had a magnitude of 1 (Fig. 4C, t = 0 h, triangle) whereas a vector from a sample taken from the middle of the movie had a magnitude of approximately 0.6 (Fig. 4C, t = 3 h, square). A summary of the results for the three groups (Fig. 4D), showed that EtOH affected the directionality of CNCC movements, reduced the magnitude of cell movements, and created greater scatter in cell orientation.

#### Analysis of lateral movement directionality

Because the angle of the mean vectors is an average of all the motion vectors in the OF motion field (Fig. 4) this measurement masks the lateral cell movements given by the angles of left and right mean vectors. Thus, we next analyzed the directionality of cell movements within the left and right side of the motion field (Fig. 5; blue = cell movements in the left half of the image, red = cell movements in the right half of image). In the control group (Fig. 5A), both left and right image fields showed a symmetrical distribution of vectors. Cells in the left image field showed directed movement between 0° and −90° and cells in the right image field show directed movement between 0° and 90° over time (Fig. 5A). Cells in both the left and right image fields showed increasing magnitude of movements during the first three hours of migration (Fig. 5A, triangles vs. squares). In contrast to the controls, cells in the 100 mM EtOH treated group (Fig. 5B) showed less symmetrical cell movements. The initial vector of the left field of cells showed movement toward the right side of the field, given by the position of the first vector at approximate 10° (Fig. 5B, blue triangle, t = 0 h). Subsequently these left image field cells showed net movements to the left at −30° (Fig. 5B, blue square, t = 3 h) and posteriorly (−120°) by the end of the migration (Fig. 5B, blue circle, t = 6 h). The right image field cells showed a similar pattern: they started on the left side of the field (−10°), then moved right (30°) and finished further right (90°) (Fig. 5B, red triangle, square and circle). The magnitude of movement was reduced during the first 3 hours of migration in both left and right image fields (Fig. 5B, magnitude differences between triangles and squares). The 200 mM EtOH treatment group (Fig. 5C) showed a similar asymmetrical distribution of cell movements between the left and right image fields with the left image field cells orienting initially towards the right between 0–30° (Fig. 5C, blue triangle and consecutive vectors), then moving left (−30°, Fig. 5C, blue square) and orienting posteriorly (−120°, Fig. 5C, blue circle). The right image field cells initiated movements toward the left image field around 15° (Fig. 5C, red triangle), then moved to the right (Fig. 5C, vectors between red triangle and square, 0°-30°) and continued moving in this direction (Fig. 5C, red circle, −90°). Similarly to the 100 mM EtOH treatment group, the magnitude of cell movements on both sides of the field decreased during the first 3 hours of migration (differences in the vector magnitudes between the triangles and squares vectors, Fig. 5C). Thus EtOH affects the directionality of CNCC cell movements enhancing left-right asymmetries relative to the midline of the developing neural tube.

### Affine Transformation

**Figure 6:**
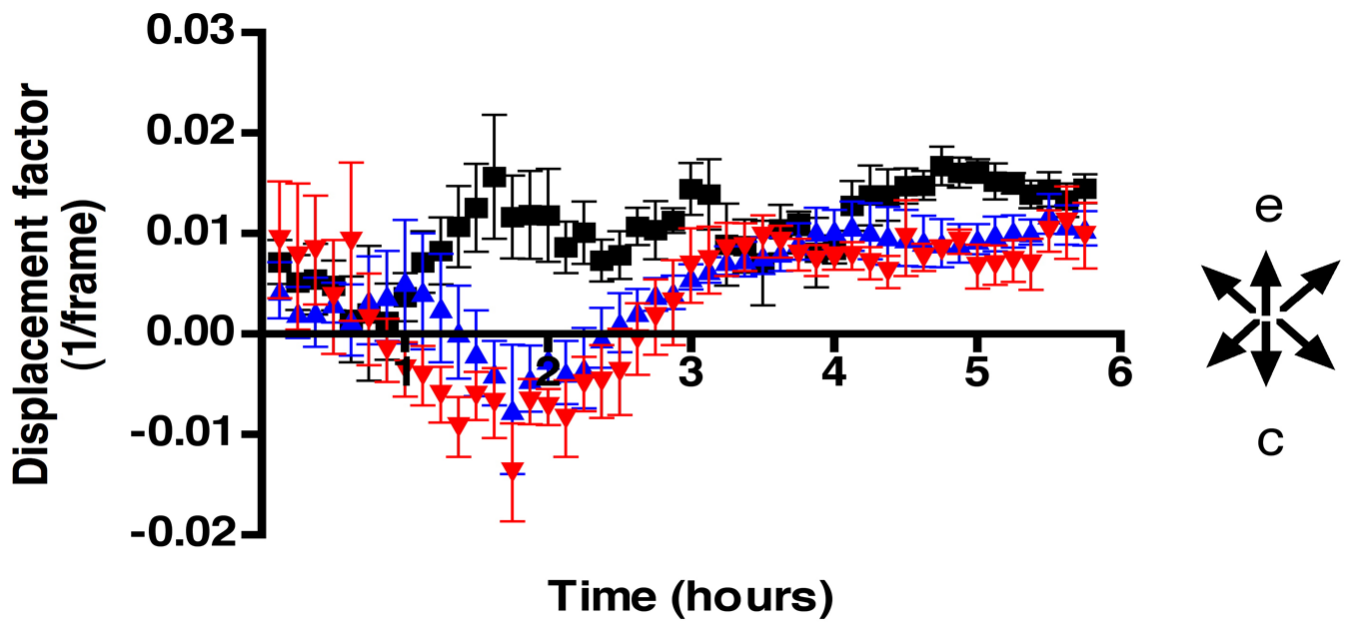
Analysis of movements by Affine transformation show that EtOH treatment affects expansion contraction movements. Affine transformation data from the OF analysis were averaged and represented over time as net displacement reflecting expansive-contractive cell movements. Positive Y values are expansive (e) cell movements and negative Y values are contractive (c) cell movements. Error bars = SEM.

The OF data were fit to an Affine transformation to obtain a measure of concerted movement through the extraction of information relative to radial expansive-contraction cell movements over time. Initially, all three groups (Fig. 6), control (black squares), 100 mM EtOH (blue triangles) and 200 mM EtOH (red triangles) treated groups showed little net displacement, reflecting low expansive or contractive cell movements. Between the first and second hour the control group increased its expansive cell movements (positive displacement values), whereas the treated embryos showed increasing contractive cell movements (negative displacement values). By the second and third hour, the treated embryos changed from contractive to expansive cell movements but their net expansive movement was less than that observed in controls (fourth to sixth hour). Data were binned by time periods, and the variance analysis (ANOVA) at each time point yielded significant differences between groups during the second, third, fifth, and sixth hours (Table 1).

**Figure 7:**
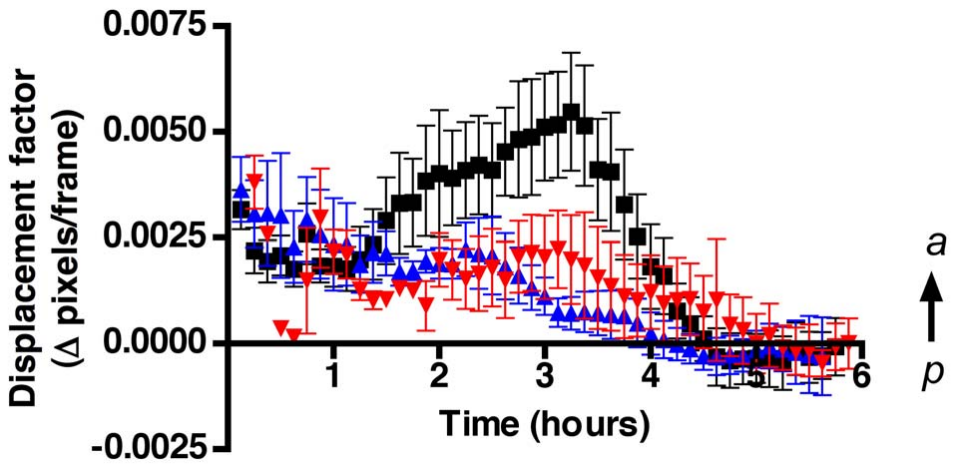
EtOH treatment affects concerted migration. Affine transformation data from the OF analysis were averaged and represented over time as net displacement reflecting coordinated posterior (p) –anterior (a) cell movements. Positive Y values represent anterior movements. Error bars = SEM.

In addition to radial expansive/contractive movements the affine transformation can extract information on vertical movements in a position-independent manner, thus evaluating concerted movements in an anterior direction (Fig. 7). As was observed in the expansive-contraction cell movements, initially all three groups (Fig. 7), control (black squares), 100 mM EtOH (blue triangles) and 200 mM EtOH (red triangles) showed little net movement in the anterior direction. In the second hour the control group showed a sharp increase in anterior movements (Fig. 7, black squares), this difference continued until hour four when the controls showed reduced anterior movement, presumably as they approached their target regions. The ANOVA analysis showed that the groups were significantly different from the second through the third hour and in the fifth hour (Table 1). The Affine transformation data revealed that EtOH altered the pattern of CNCC migration by reducing expansive cell movements thus affecting the coordination of cell movements in an anterior direction, which is in agreement with the results obtained in the analysis of angles (Fig. 4 and 5).

### Analysis of Cell Death in the Craniofacial Area

**Figure 8:**
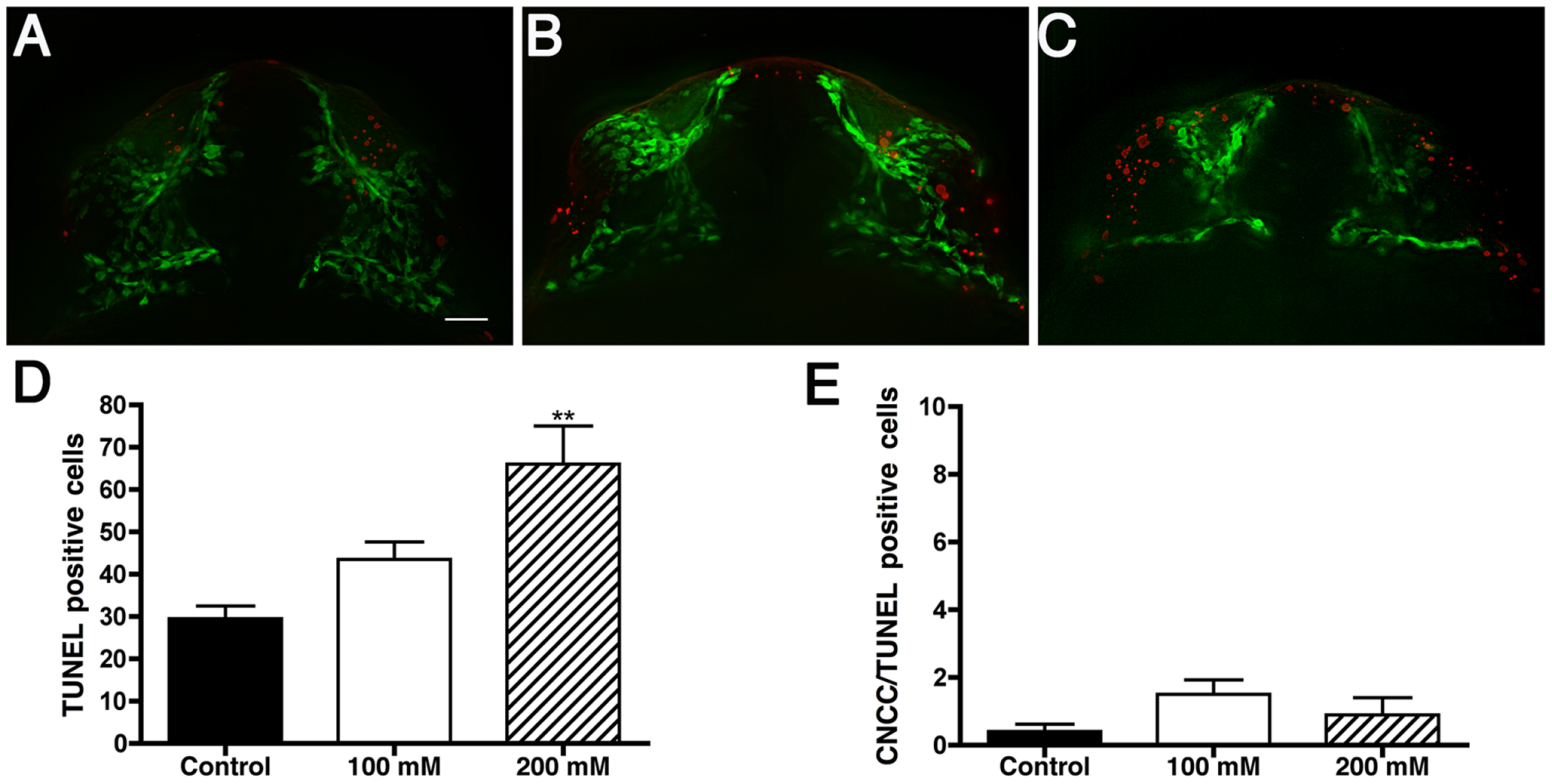
Ethanol increases non-specific cell death. Fluorescent images showing cell death (red) and CNCC (green) in A) Control, B) 100 mM EtOH and C) 200 mM EtOH treated *sox10:EGFP* 24 hpf embryos. D) Variance analysis reveals a significant increase in the number TUNEL positive cells (p<0.0007; n = 10 per treatment) throughout the craniofacial area. E) The number of CNCC positive for both EGFP and TUNEL are: control = 0.4 cells, 100 mM = 1.5 cells, and 200 mM = 0.9 cells. Error bars = SEM. Frontal views: dorsal to the top. Scale bar = 20 µm.

As a control for potential negative effects of Sox10:EGFP expression, we analyzed cell death using TUNEL in control (Fig. 8A, red), 100 and 200 mM EtOH treatments (Fig. 8B, C, red) *sox10:EGFP* embryos. Because DNA fragmentation is a late event in cell death where TUNEL positive cells start to appear at 3 hours after cytotoxic events, peaking at nine hours [25], we used 24 hpf (26+ somites) as the time to quantify potential EtOH induced cell death. Variance analysis of TUNEL experiments revealed a significant increase (p<0.0007 n = 10/treatment) in the number of TUNEL positive cells throughout the craniofacial area (Fig. 8D). Post-hoc analysis also showed significant differences between control versus 200 mM EtOH treatment (p<0.001) and 100 mM versus 200 mM EtOH treatment (p<0.05). There was also an increase in TUNEL positive cells throughout the embryo (data not shown), which is in agreement with previous literature. Nevertheless, we observed no statistically significant co-localization of TUNEL positive cells with Sox10:EGFP positive CNCC. Indeed, the average number of co-localizing cells for control embryos was 0.4 cells, 1.5 cells for 100 mM, and 0.9 cells for 200 mM (Fig. 8E) (p>0.1736, ANOVA). Thus our results suggest that survival of Sox10:EGFP expressing cells is unaffected by ethanol exposure.

## Discussion

The use of OF for image processing to quantify CNCC population migration patterns *in vivo* enabled us to compare quantitatively CCNC migration in normal and EtOH exposed animals. Overall the migration patterns of CNCC that migrate dorsal to the eye in EtOH exposed embryos appeared more disorganized and reduced; thus, sub-lethal concentrations of EtOH change the migration patterns of the CNCC. The resulting morphological craniofacial phenotypes we observed in the EtOH exposed embryos (Boric, Couve, and Whitlock, unpublished) are in agreement with previous studies showing that EtOH treatment results distinct reproducible phenotypes [22], [26]–[31]. Of particular interest are the results obtained with lower concentrations of EtOH exposure because they most likely correspond this model organism’s version of craniofacial phenotypes of fetal alcohol syndrome (FAS) patients that are caused by exposure to sub-lethal concentrations of EtOH in developing humans. The majority of embryos exposed the 100 mM and 200 mM live to adulthood, yet show behavioral defects (Boric, Stephenson & Whitlock unpublished). The challenge has been how to quantify these sub-lethal defects that account for FAS. The advantages of the population wide analysis of CNCC migration are that this analysis reveals subtle differences in cell movements and these differences can be quantified in a rigorous manner.

### Cell Movements

The greatest strength of the OF analysis is that we were able to quantify defects in the migration of CNCC that to date has only been described qualitatively. Observing the time-lapse movies of CNCC migration one can see that the EtOH exposed embryos are different but until now we lacked a rigorous protocol to analyze this difference in terms of symmetry of movements across the midline, as well as persistence directionality of the population of cells. Initial analysis (Fig. 2) of the direction and magnitude of the movement of the CNCC population revealed differences between the left and right visual fields, representing the left and right halves of the head. The differences were small in the control but greatly exaggerated in the EtOH exposed embryos. It is well known that left-right asymmetry in animals exists as part of genetically controlled developmental programs such as those underlying heart looping [32]. More interestingly, for our results, are the common left-right asymmetries that have been reported in faces of normal humans [33], [34]. This uncoupling of left and right symmetry results in mild asymmetries, called Facial Asymmetries (FA), that arguably contribute to physical attractiveness of the individual [35] and may signal the health state of the individual as reflected by developmental stressors [36]. EtOH is a known developmental stressor with prenatal exposure resulting in brain and facial defects in humans [37]. Recently an analysis of FA in children with prenatal exposure to alcohol has shown that in addition to the previously characterized FAS facial characteristics, there exists significant level of directional asymmetry [38]. In our analysis of magnitude of displacement in left and right cell fields (Fig. 2) as well as the direction (color hue) and magnitude (color intensity) of the displacement, all become increasingly different following EtOH exposure. Further analysis of the cells in the left and right cell fields (Fig. 5) shows a lack of symmetry between the left and right side, as evidenced by the uncoupling of the mirror image movement seen in controls. Our observations correlate with recent observations where prenatal exposure to alcohol in humans (FAS) results in structural features around the eyes being shifted toward the left cell field and midline structures to the right [38]. Thus, the method presented here has the ability to quantify the subtle changes underlying normal and ethanol-induced facial asymmetry.

The overall loss of directionality, in part evidenced by the asymmetry between the left and right cell fields, affects the net movement of the cell fields. Analysis of the angles and magnitude of the movements (Fig. 4) revealed a decrease in directionality of the movement. Further analysis, applying the affine transformation (Figs. 6, 7), shows a decrease in expansive-contractive cell movements toward the anterior of the head suggesting a loss of coordinated cell movements, or directionally persistent migration, as described at the single cell level where cells form lamellipodia and stabilize connections at their leading edge [39]. Directional persistence is divided into short scale directional persistence whose dynamics are driven by lamellipodial contact, and long time scale directional persistence where adhesion interactions enhance contact through expression of growth factors and adhesion molecules [40], [41]. Given the nature of our analysis, on the population level, it is not possible to differentiate the potential contributions to the observed defects from changes that occurred over short vs. the long time scale, although the literature supports a role for loss of specific cell adhesion molecules as one of the causes of ethanol induced defects in cell migration [41], [42].

### Cell Death in the Developing Embryo

We showed that EtOH significantly increased cell death throughout the craniofacial area; however, no increase in cell death was observed within Sox10:EGFP positive CNCC. These results suggest that the expression of EGFP under control of the *sox10* promoter does not have deleterious effects in the presence of EtOH. Cell death in the frontonasal process, which is mainly derived from CNCC, and the forebrain is one of the most accepted mechanism underlying FAS craniofacial and nervous system phenotypes [43]. Primary cultures of CNCC from mouse [13] and chick [12] show increased cell death in EtOH treated cultures. Additionally, experiments performed *in ovo*, where EtOH was injected directly into the chick egg, also reported increased cell death of CNCC [9], [11]. The discrepancy between our findings and those in the literature could be explained by *in ovo* injections where the EtOH is introduced directly into the embryos, in contrast to our method where EtOH is introduced in the embryo medium. In this situation, EtOH has to cross the chorion and enter the embryo, and the chorion causes a decrease in about 80% of the external EtOH concentration (data unpublished). Finally, the CNCC we have followed are those that are under genetic control of *sox10*, which are known to be the non-ectomesenchymal subset of CNCC, thus the previously reported cell death in CNCC could be in the ectomesenchymal population. Nevertheless, the lack of cell death within Sox10:EGFP positive cells in the craniofacial area suggests that increased cell death of these progenitors in some model systems is not the only mechanism underlying EtOH induced craniofacial defects.

### CNCC Migration as a Population

We were able to characterize the migration of CNCC populations in the living embryos and use EtOH as a representative environmental toxicant to better understand detrimental effects on CNCC migration. The analysis of migration on a population-wide basis was very important because it has been shown that CNCC migrate “collectively” and that cooperation between individual cells contributes to the overall directionality of the group [1]. The majority of the available imaging methods use single cell trajectories that are measured and can be accumulated to “create” a population. The results obtained with these approaches can be biased, are labor intensive, and the results often lose important information on dynamics of cell populations during migration. We adapted the image processing method “Optical Flow” [16], [23] which can be easily executed in a Matlab platform and yields several quantifiable and straightforward parameters of group migratory behaviors, allowing us to quantify environmentally induced defects in CNCC migration. Our analysis has uncovered a previously undescribed effect of ethanol on early development: subtle defects in left-right asymmetry of CNCC migration possibly underlying the enhanced facial asymmetries recently reported in the literature. Because FA has been suggested to reflect the effects of developmental stressors, the EtOH effects on left-right migration may be the most subtle defect in FAS. Thus the face, a feature to which humans are exquisitely sensitive and is created through migratory movements of CNCC, is a testimony of early development: CNCC can recover from exposure to environmental insults but the history is etched on the face through the struggles of the cells.

## Supporting Information

Video S1 **Time-lapse movie of CNCC migration in control embryo.** Example of CCNC expressing Sox10:EGFP (green) visualized in a living embryo. View is frontal with dorsal to the top of the page. The movie was initiated at the 6–8 somite stage and continued until the 20–22 somite stage. See Figure 2A for further details. Anterior movements are toward the bottom of the page.
(AVI)

Video S2 **Time-lapse movie of CNCC migration in embryo treated with 100 mM EtOH.** Example of CNCC expressing Sox10:EGFP (green) in a living embryo treated with 100 mM EtOH. View is frontal with dorsal to the top of the page. CNCC show delayed migration relative to the control. See Figure 2B for further details. Anterior movements are toward the bottom of the page.
(AVI)

Video S3 **Time-lapse movie of CNCC migration in embryo treated with 200 mM EtOH.** Example of CNCC expressing Sox10:EGFP (green) in a living embryo treated with 200 mM EtOH. View is frontal with dorsal to the top of the page. The CNCC migrate only to the posterior edge of the eye and do not reach the eye at 20–22 somites. See Figure 2C for further details. Anterior movements are toward the bottom of the page.
(AVI)
